# Association of Blood Pressure with Fasting Blood Glucose Levels in Northeast China: A Cross-Sectional Study

**DOI:** 10.1038/s41598-018-26323-6

**Published:** 2018-05-21

**Authors:** Yaogai Lv, Yan Yao, Junsen Ye, Xin Guo, Jing Dou, Li Shen, Anning Zhang, Zhiqiang Xue, Yaqin Yu, Lina Jin

**Affiliations:** Epidemiology and Biostatistics, School of Public Health, No. 1163 Xinmin Street, Jilin University, Changchun, Jilin 130021 China

## Abstract

Hypertension and diabetes mellitus (DM) have become major public health issues, and previous studies have shown that there is an association between hypertension and DM. However, there is a lack of detailed information about this association. This study aimed to explore how different blood pressure (BP) levels were associated with fasting blood glucose (FBG) levels. A cross-sectional survey with adults aged 18 to 79 years was conducted in Jilin Province, China in 2012. Lambda-mu-sigma (LMS) was used to preliminarily explore the associations of BP with FBG. Quantile regression (QR) was performed to identify the specific associations by adjusting for confounding factors. The distributions of systolic blood pressure (SBP) (*χ*^2^ = 710.76, *P* < 0.001) and diastolic blood pressure (DBP) (*χ*^2^ = 460.20, *P* < 0.001) were different according to gender. LMS showed that the associations of BP with FBG became stronger when the FBG levels were close to 5.6 mmol/L. QR showed that FBG was positively associated with SBP (*P*_30_ to *P*_90_) and DBP (*P*_20_ to *P*_90_) in males. In females, FBG was positively associated with SBP from only *P*_85_ to *P*_90_. In summary, FBG was positively associated with BP in a gender-dependent manner.

## Introduction

Currently, both hypertension and diabetes mellitus (DM) are serious global public health issues^1–3^, and previous studies have shown that there is an association between hypertension and DM^4,5^. On the one hand, patients with DM are at increasing risk of developing hypertension compared to those without DM^6,7^. On the other hand, the association between hypertension and DM may increase the risk of the co-occurrence of these two diseases, and then the interaction of these two diseases could lead to new public health issues, such as increasing the risk of the development of stroke^8^. Moreover, among DM patients with hypertension, insulin resistance is an independent risk factor for ischaemic cerebral infarction, especially lacunar infarction^9^. Furthermore, the combination of hypertension and DM can significantly increase circadian rhythm abnormalities and target organ damage^10^.

Many studies have focused on the association between hypertension and DM, and most of them have treated blood pressure (BP) and fasting blood glucose (FBG) as categorical variables (transforming the data into “normal” and “abnormal”)^4,11^. However, the occurrence and development of hypertension is a continuous and long-term process. BP, a sensitive index for diagnosing hypertension, can reflect the progression of hypertension to some extent. Yet no study has fully reflected the whole distribution of BP, and studies on the association of BP with FBG have been inadequate^12^. There still exists a huge gap in our understanding on the association of BP with FBG.

In view of the above facts, it was a challenge to explore the association of BP with FBG by using the whole distribution of BP rather than the average BP with conventional methods (such as logistic regression). Fortunately, the lambda-mu-sigma (LMS) and quantile regression (QR) methods are well suited to solving this problem. Therefore, in this study we aimed to explore the associations of different levels of BP with FBG by using LMS and QR methods based on a cross-sectional study in Jilin Province, China.

## Results

### Descriptive characteristics of participants by gender

In total, 11,878 subjects were enrolled in the study, with 5,571 males and 6,307 females. As shown in Table 1, BMI, WC, SBP, DBP and FBG were all significantly higher in males than those in females (*P* < 0.05), and females were older than males (*P* < 0.05). There were significant differences between males and females in the distributions of alcohol consumption, smoking status and dyslipidaemia (*P* < 0.05) as well.

**Table 1:** Descriptive characteristics of participants by gender.

Variables	Male (n = 5571)	Female (n = 6307)	*Z*/*χ*^2^	*P*-value
Age^a^	44.0 (34.0, 54.0)	45.0 (37.0, 54.0)	−5.2	<0.001
BMI^a^	23.7 (21.3, 26.2)	23.3 (21.1, 25.7)	−5.5	<0.001
WC^a^	83.0 (75.8, 90.0)	78.0 (71.0, 85.0)	−26.0	<0.001
SBP^a^ (mmHg)	128.0 (119.0, 139.0)	120.0 (110.0, 132.0)	−27.7	<0.001
DBP^a^ (mmHg)	80.0 (73.0, 87.0)	75.0 (69.0, 81.0)	−24.9	<0.001
FBG^a^	5.1 (4.6, 5.7)	4.9 (4.4, 5.4)	−15.7	<0.001
Dyslipidaemia^b^			141.0	<0.001
No	3489 (62.6)	4592 (72.8)		
Yes	2082 (37.4)	1715 (27.2)		
Alcohol consumption^b^			3064.4	<0.001
No	2296 (41.2)	5625 (89.2)		
Yes	3275 (58.8)	682 (10.8)		
Smoking status^b^			3465.4	<0.001
Never smoker	1905 (34.2)	5468 (86.7)		
Former smoker	614 (11.0)	162 (2.6)		
Current smoker	3052 (54.8)	677 (10.7)		

^a^Presented as the median (inter-quartile range); ^b^presented as n (%).

BMI: body mass index; WC: waist circumference; SBP: systolic blood pressure; DBP: diastolic blood pressure; and FBG: fasting blood glucose.

### Distribution of BP in males and females

**Figure 1:**
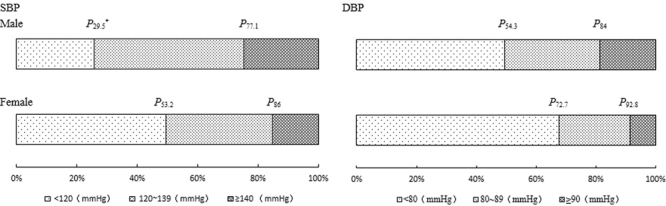
Distribution of BP in males and females. ^*^P_x_: percentile x; SBP: systolic blood pressure; and DBP: diastolic blood pressure.

Figure 1 shows the distributions of SBP (*χ*^2^ = 710.76, *P* < 0.001) and DBP (*χ*^2^ = 460.20, *P* < 0.001) by gender, and there were significant differences between males and females (hypotension was merged into normal BP due to the small population). Table 2 shows the quantiles of SBP and DBP, and the levels of BP were different according to gender. Therefore, the following results were analysed separately in males and females.

**Table 2:** Quantiles of blood pressure (SBP & DBP) by gender.

Variable	Quantiles										
	*P*_10_	*P*_20_	*P*_30_	*P*_40_	*P*_50_	*P*_60_	*P*_70_	*P*_75_	*P*_80_	*P*_85_	*P*_90_
Male											
SBP (mmHg)	111	117	120	124	128	131	136	139	142	146	151
DBP (mmHg)	68	71	74	77	79	82	85	86	88	90	94
Female											
SBP (mmHg)	102	108	112	116	119	123	128	131	135	139	146
DBP (mmHg)	63	67	70	72	74	77	79	81	83	85	89

Numbers in the Table are blood pressure in different quantiles.

### Results of LMS analysis

**Figure 2:**
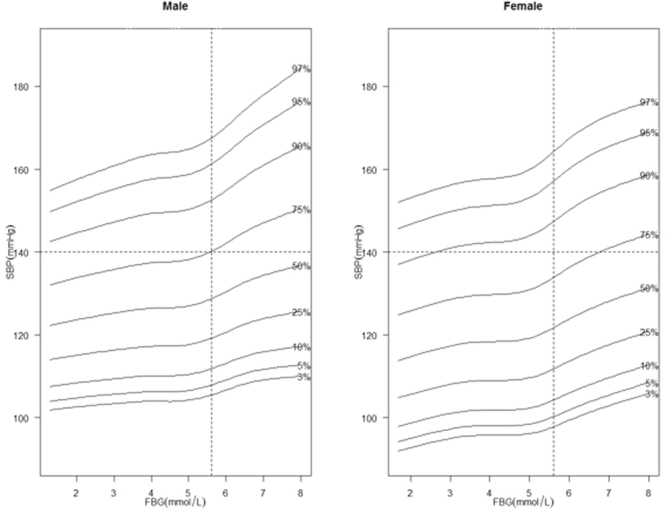
Smoothed SBP percentile curves for males and females.

**Figure 3:**
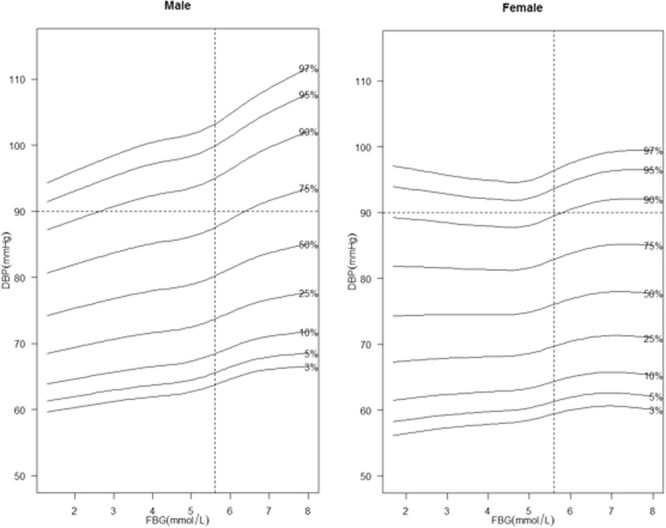
Smoothed DBP percentile curves for males and females.

The smoothed percentile curves of SBP and DBP in males and females are shown separately in Figs 2 and 3. The shapes of the curves were different according to gender, and all the percentiles of BP in males were substantially and consistently higher compared with those in females. Moreover, the associations of BP with FBG became stronger when the FBG levels were close to 5.6 mmol/L.

### Results of the QR model in males

The coefficients of FBG in different quantiles of SBP and DBP are shown separately in Tables 3 and 4. Table 3 shows that there were positive associations of SBP with FBG, and the coefficients increased from *P*_10_ to *P*_90_ (model 1). In addition, FBG was positively associated with SBP from *P*_30_ to *P*_90_ after adjusting for age, BMI, WC, smoking status, alcohol consumption and dyslipidaemia (model 2). Table 4 shows that FBG was positively associated with DBP from *P*_20_ to *P*_90_ (model 1). In addition, FBG was still positively associated with DBP from *P*_20_ to *P*_90_ after adjusting for age, BMI, WC, smoking status, alcohol consumption and dyslipidaemia (model 2).

**Table 3:** Quantile regression coefficients [95% *CI*] between FBG and SBP in males.

Model	*P*_10_	*P*_20_	*P*_30_	*P*_40_	*P*_50_	*P*_60_	*P*_70_	*P*_75_	*P*_80_	*P*_85_	*P*_90_
Model 1	0.959^*^ [0.542,1.480]	1.154^*^ [0.404,1.655]	1.429^*^ [0.700,2.569]	1.795^*^ [1.047,2.815]	2.245^*^ [1.212,3.074]	2.500^*^ [1.897,3.380]	2.963^*^ [1.978,3.630]	3.077^*^ [2.242,3.947]	2.941^*^ [1.947,5.840]	3.077^*^ [1.990,5.136]	3.548^*^ [2.183,4.527]
Model 2	0.381^*^ [0.114,0.741]	0.341 [−0.050,0.715]	0.744^*^ [0.148,1.143]	0.616^*^ [0.085,1.008]	0.581^*^ [0.262,1.353]	0.895^*^ [0.353,1.327]	0.991^*^ [0.367,1.490]	0.857^*^ [0.378,1.718]	1.039^*^ [0.339,1.893]	1.595^*^ [0.538,2.060]	1.617^*^ [0.527,2.214]

^*^P < 0.05.

*CI*: confidence interval.

Model 1, without adjustments for the confounding factors.

Model 2, adjusted for age, BMI, WC, smoking status, alcohol consumption and dyslipidaemia.

**Table 4:** Quantile regression coefficients [95% *CI*] between FBG and DBP in males.

Model	*P*_10_	*P*_20_	*P*_30_	*P*_40_	*P*_50_	*P*_60_	*P*_70_	*P*_75_	*P*_80_	*P*_85_	*P*_90_
Model 1	0.930 [−0.180,1.058]	1.000^*^ [0.608,1.274]	1.176^*^ [0.628,1.486]	1.190^*^ [0.762,1.686]	1.146^*^ [0.758,1.806]	1.071^*^ [0.830,1.801]	1.429^*^ [0.580,2.290]	1.538^*^ [0.680,2.640]	1.600^*^ [0.708,3.352]	1.818^*^ [0.957,2.578]	2.143^*^ [1.407,2.774]
Model 2	0.291 [−0.040,0.555]	0.477^*^ [0.186,0.652]	0.368^*^ [0.170,0.661]	0.357^*^ [0.039,0.663]	0.310^*^ [0.053,0.608]	0.275^*^ [0.037,0.646]	0.553^*^ [0.092,0.829]	0.464^*^ [0.165,0.737]	0.281^*^ [0.038,0.892]	0.603^*^ [0.140,1.140]	0.536^*^ [0.025,1.533]

^*^P < 0.05.

*CI*: confidence interval.

Model 1, without adjustments for the confounding factors.

Model 2, adjusted for age, BMI, WC, smoking status, alcohol consumption and dyslipidaemia.

### Results of the QR model in females

The coefficients of FBG in different quantiles of SBP and DBP are shown separately in Tables 5 and 6. As shown in Table 5, FBG was positively associated with SBP from *P*_10_ to *P*_90_ (model 1), whereas FBG had positive associations with SBP from only *P*_85_ to *P*_90_ after adjusting for age, BMI, WC, smoking status, alcohol consumption and dyslipidaemia (model 2). As shown in Table 6, there were positive associations of DBP with FBG from *P*_10_ to *P*_30_ and from *P*_75_ to *P*_90_ (model 1). Yet the associations of DBP with FBG were not statistically significant after adjusting for age, BMI, WC, smoking status, alcohol consumption and dyslipidaemia (model 2).

**Table 5:** Quantile regression coefficients [95% *CI*] between FBG and SBP in females.

Model	*P*_10_	*P*_20_	*P*_30_	*P*_40_	*P*_50_	*P*_60_	*P*_70_	*P*_75_	*P*_80_	*P*_85_	*P*_90_
Model 1	1.176^*^ [0.453,2.573]	1.765^*^ [0.795,2.241]	1.852^*^ [1.068,2.265]	1.907^*^ [1.281,3.166]	2.250^*^ [1.320,3.165]	2.500^*^ [1.598,3.422]	2.333^*^ [1.888,3.583]	2.000^*^ [1.528,3.550]	1.875^*^ [1.234,3.671]	2.745^*^ [0.767,3.720]	2.857^*^ [1.926,6.713]
Model 2	0.267 [−0.210,0.585]	0.492 [−0.051,0.879]	0.236 [−0.101,0.735]	0.121 [−0.285,0.668]	0.195 [−0.397,0.641]	0.016 [−0.604,0.704]	0.234 [−0.349,0.637]	0.134 [−0.458,0.649]	0.123 [−0.394,0.581]	0.356^*^ [0.027,1.259]	0.272^*^ [0.083,1.288]

^*^P < 0.05.

CI: confidence interval.

Model 1, without adjustments for the confounding factors.

Model 2, adjusted for age, BMI, WC, smoking status, alcohol consumption and dyslipidaemia.

**Table 6:** Quantile regression coefficients [95% *CI*] between FBG and DBP in females.

Model	*P*_10_	*P*_20_	*P*_30_	*P*_40_	*P*_50_	*P*_60_	*P*_70_	*P*_75_	*P*_80_	*P*_85_	*P*_90_
Model 1	0.690^*^ [0.345,0.950]	0.556^*^ [0.257,0.687]	0.435^*^ [0.332,0.897]	0.123 [−0.085,0.837]	0.556 [−0.138,1.089]	0.667 [−0.221,1.192]	0.571 [−0.144,1.780]	0.909^*^ [0.321,1.580]	0.769^*^ [0.198,1.631]	0.794^*^ [0.433,2.515]	0.909^*^ [0.163,2.866]
Model 2	0.086 [−0.484,0.214]	−0.022 [−0.237,0.194]	−0.130 [−0.291,0.129]	−0.154 [−0.375,0.083]	−0.160 [−0.386,0.151]	−0.022 [−0.478,0.273]	0.016 [−0.268,0.318]	−0.016 [−0.176,0.408]	0.037 [−0.269,0.508]	0.083 [−0.320,0.447]	0.142 [−0.324,0.615]

^*^P < 0.05.

*CI*: confidence interval.

Model 1, without adjustments for the confounding factors.

Model 2, adjusted for age, BMI, WC, smoking status, alcohol consumption and dyslipidaemia.

## Discussion

The findings of this study could be summarized as follows. First, there were positive associations of BP with FBG, and the associations were different according to gender. FBG was associated with BP in almost all quantiles in males, while in females, only SBP in high quantiles had positive associations with FBG. Second, the associations of BP with FBG became stronger when the FBG levels were close to 5.6 mmol/L.

In our study, we found that FBG was positively associated with BP, which was consistent with the results of similar previous studies^13,14^. The possible mechanism is that as the FBG level increases, hyperglycaemia with insulin resistance, overweight and metabolic disorders could alter the rennin-angiotensin system (RAS), then leading to an effect on BP^15,16^.

In addition, we found that the associations of BP with FBG were different in males and females; FBG was positively associated with both SBP and DBP in almost all quantiles in males, whereas FBG had positive associations with only SBP in the high quantiles in females. The finding implied that the positive associations of BP with FBG were more notable in males than in females. There are several reasons for these associations. First, the risk of hypertension in females is lower than that in males^17^. Second, evidence has shown that the lower endogenous oestrogen levels found in males might be related to higher insulin resistance when compared with females^18^, which means that the same FBG levels in males and females may lead to different physiological effects due to different levels of insulin resistance. Thus, the finding might suggest that control of one’s FBG level should be more emphasized and strengthened among males than females, except females with high SBP.

The cut-off value for the definition of impaired fasting glycaemia (IFG) is still controversial at present; the American Diabetes Association (ADA) Expert Committee reduced the cut-off value of IFG to 5.6 mmol/L in 2003, while the World Health Organization (WHO) retained the previous cut-off value of IFG (6.1 mmol/L)^19^. Further, IFG has been shown as an independent risk factor for hypertension^20,21^; however, whether the FBG level begin to become a potential risk factor for hypertension when it is close to 5.6 mmol/L is uncertain. Fortunately, our study showed that the positive associations of BP with FBG became stronger when FBG levels were close to 5.6 mmol/L, which implied that FBG level might begin to become a potential risk factor for hypertension when it was close to 5.6 mmol/L. A possible explanation is that the body may develop hyperinsulinaemia before FBG level reaches 6.1 mmol/L, and the presence of hyperinsulinaemia could directly contribute to an elevation in BP by increasing renal sodium retention^22^. Therefore, having awareness of controlling FBG levels when they approach 5.6 mmol/L might have potential benefits, such as early control of BP. Furthermore, having awareness of controlling FBG levels might also assist in preventing the development of co-occurrence of hypertension and DM to some extent.

Some limitations should be noted in this study. First, participants in the study were from Jilin Province in Northeast China, and the results may not be generalizable to other areas. Second, some of the subjects were excluded because they did not suit the purpose of the study and/or they were with incomplete data on BP, thus, selection biases might be present. Third, other confounding factors (such as genes) may have impacted the results. Finally, the data were obtained from a cross-sectional survey; therefore, further studies are needed to explore the associations in a longitudinal setting.

## Conclusions

There were positive associations of BP with FBG, and these associations were different according to gender. FBG was associated with BP in almost all quantiles in males, yet in females, FBG had positive associations with only SBP in the high quantiles. Moreover, the associations of BP with FBG became stronger when the FBG levels were close to 5.6 mmol/L.

## Methods

### Study population

**Figure 4:**
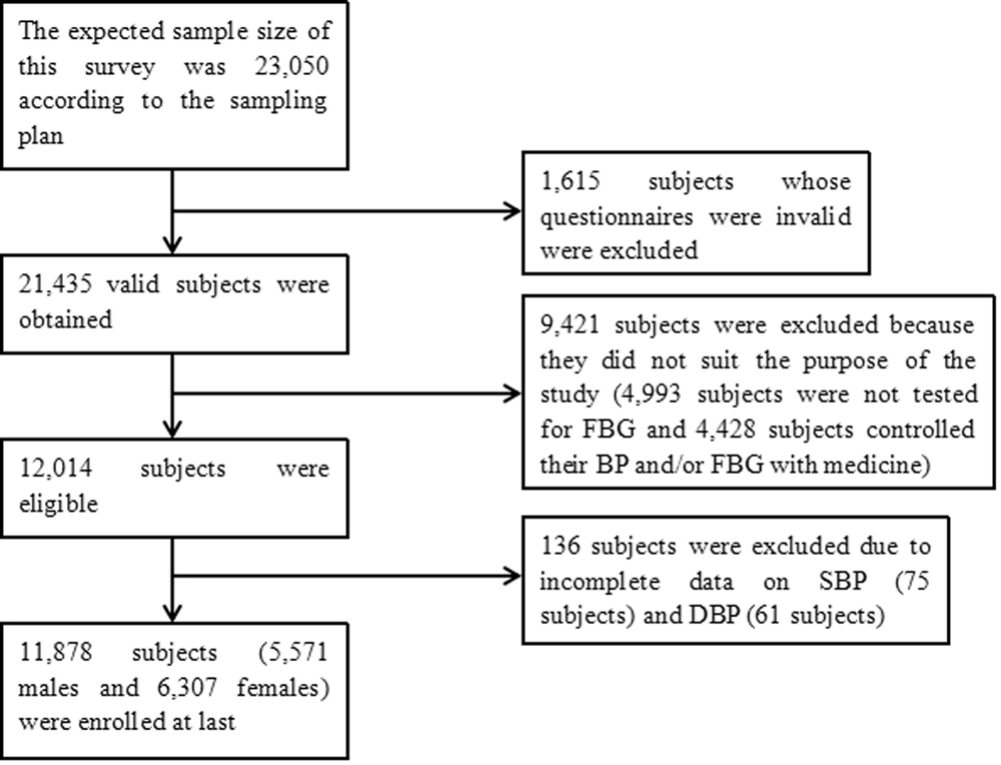
Flow diagram of the study design and participant selection process for this study.

Data was derived from a cross-sectional study of chronic disease conducted by the Jilin University School of Public Health and the Jilin Department of Health in Jilin Province of China in 2012. In this study, a total of 23,050 subjects who had lived in Jilin Province for more than 6 months and were 18–79 years old were selected through multistage stratified random cluster sampling^23^. First, 1,615 subjects whose questionnaires were invalid were excluded. Second, 9,421 subjects were excluded because they did not suit the purpose of the study (4,993 subjects were not tested for FBG and 4,428 subjects controlled their BP and/or FBG level with medicine). Third, 136 subjects were excluded due to incomplete data on systolic blood pressure (SBP) (75 subjects) and diastolic blood pressure (DBP) (61 subjects). Finally, 11,878 subjects were included in this study (Fig. 4). All participants provided written informed consent, and the study was approved by the Institutional Review Board of the Jilin University School of Public Health. In addition, all analyses were performed in accordance with the relevant guidelines and regulations.

### Data Collection and Measurement

The data in this study were collected by investigators who had been uniformly trained. The information included demographics (gender, age), health-related behaviours (smoking status, alcohol consumption), anthropometric measurements (SBP, DBP and waist circumference (WC), etc.) and biochemical tests (FBG, total cholesterol (TC) and triglycerides (TG), etc.). BP was measured using a mercury sphygmomanometer by trained professionals, and the subjects were required to have rested for at least 5 min before BP was measured. Two readings of SBP and DBP were recorded, and the average values were used for the data analysis. If the first two measurements differed by more than 5 mmHg, additional readings were taken. FBG levels were measured by the Bai Ankang fingertip blood glucose monitor (Bayer, Leverkusen, Germany).

Serum lipid levels (TG, TC, low-density lipoprotein cholesterol (LDL-C), high-density lipoprotein cholesterol (HDL-C)) were measured by the MODULE P800 biochemical analysis machine (Roche Co., Ltd., Shanghai, China) in the morning after participants had fasted for 10 or more hours overnight. The participants’ height and weight were measured according to a standardized protocol with the participants wearing clothing but no shoes, and WC was measured at the midpoint between the lowest rib margin and the iliac crest with the participants standing. Body mass index (BMI) was calculated using the following formula: BMI = Weight (kg)/Height (m)^2^.

### Assessment Criteria

Hypertension was defined as resting SBP ≥ 140 mmHg and/or DBP ≥ 90 mmHg; high-normal BP was defined as SBP 120–139 mmHg or DBP 80–89 mmHg; and normal BP was defined as SBP < 120 mmHg and DBP < 80 mmHg, with no antihypertensive treatment^24^. Smoking status was categorized into never smoker (had never smoked cigarettes or had smoked fewer than 100 cigarettes in one’s lifetime), former smoker (had smoked at least 100 cigarettes in one’s lifetime but was not currently smoking), and current smoker (had smoked at least 100 cigarettes in one’s lifetime and was still smoking)^25^. Alcohol consumption was defined as consuming any type of purchased or homemade alcohol-containing beverages on average more than once per week^26^. Regarding serum lipid level, any of the following criteria was defined as dyslipidaemia: high TC: TC ≥ 6.22 mmol/L; high TG: TG ≥ 2.26 mmol/L; low HDL-C < 1.04 mmol/L; and high LDL-C ≥ 4.14 mmol/L^27^.

### Statistical Analysis

Continuous variables were presented as medians (inter-quartile range) because they did not have normal distributions. Wilcoxon rank sum tests were used to make comparisons according to gender. Categorical variables were presented as counts or percentages and compared with the Rao-Scott Chi-square test. Sex-specific percentile curves of BP with FBG were constructed by LMS method developed by Cole and Green^28^. The 3rd, 5th, 10th, 25th, 50th, 75th, 90th, 95th and 97th^29^ percentile BP curves were constructed to explore the associations of BP with FBG and to determine the general trend of those associations. QR, which is very flexible, especially for data with a heterogeneous conditional distribution, was performed to establish 2 models to explore how different BP levels were associated with FBG levels according to gender (model 2 was adjusted for age, BMI, WC, smoking status, alcohol consumption and dyslipidaemia). Meanwhile, QR was applied to build a series of regression equations in all quantiles of BP, and thus, the extreme data, such as the high BP quantiles, could be analysed^30^. R version 3.3.3 (University of Auckland, Oakland, New Zealand) was used to perform the statistical analyses. Statistical significance was set at *P*-value < 0.05.

### Data Availability

The survey was implemented by School of Public Health, Jilin University and Jilin Center for Disease Control and Prevention in Jilin Province in 2012. According to the relevant regulations, we were sorry that the data can’t be shared.
